# Phenolic Profile of Seedless *Ziziphus mauritiana* Fruits and Leaves Extracts with *In Vivo* Antioxidant and Anti‐Inflammatory Activities: Influence on Pro‐Inflammatory Mediators

**DOI:** 10.1002/cbdv.202401728

**Published:** 2024-12-12

**Authors:** Arifa Khanam, Abdullah Ijaz Hussain, Esraa Haji Mohammed, Lutfun Nahar, Hassaan A. Rathore

**Affiliations:** ^1^ Department of Chemistry Government College University Faisalabad Faisalabad 38000 Pakistan; ^2^ Department of Pharmaceutical Chemistry College of Pharmacy University of Hafr Al Batin Hafr Al Batin 39524 Saudi Arabia; ^3^ Laboratory of Growth Regulators Palacký University and Institute of Experimental Botany The Czech Academy of Sciences Šlechtitelů 27 Olomouc 78371 Czech Republic; ^4^ Department of Pharmaceutical Sciences College of Pharmacy QU Health Qatar University Doha Qatar

**Keywords:** DPPH radical scavenging, HPLC analysis, Chlorogenic acid, Inflammation, Carrageenan, CRP, IL-6, and TNF-α, Oxidative stress

## Abstract

The present study aimed to assess the antioxidant and anti‐inflammatory activities of polyphenol‐rich extracts of seedless variety of *Ziziphus mauritiana* (SZM). Reverse Phase High Performance Liquid Chromatography (RP‐HPLC) analysis of SZM leaves and fruit extracts in ethanol revealed the presence of sixteen phenolics including chlorogenic acid, *p*‐coumeric acid, gallic acid, kaempferol, rutin and quercetin. Leaf extract showed higher total phenolic and total flavonoid contents (177.6 mg/100 g and 46.2 mg/100 g) than in fruit extract (137.8 mg/100 g and 14.1 mg/100 g). The leaf extract exhibited higher DPPH radical‐scavenging activity (63.5 %) than the fruit extract (58.2 %). The anti‐inflammatory activity was evaluated on carrageenan‐induced rat model and suppression of inflammatory biomarkers (Interleukin‐6, Tumor necrosis factor‐α and CRP) were studied. The fruit extract exhibited remarkable inhibition (98.1 %) at the dose level of 500 mg/kg body weight (BW), comparable to the standard drug indomethacin (98.4 %). Both extracts suppressed the inflammatory biomarkers and more pronounced results showed by the fruit extract including CRP, IL‐6, and TNF‐α. The leaf extract demonstrated the higher antioxidant potential as evident from the superoxide dismutase, catalase, malondialdehyde, glutathione peroxidase and glutathione levels. These findings suggest that SZM leaf and fruit extracts possess potential antioxidant and remarkable anti‐inflammatory properties and can play a significant role in mitigating oxidative stress.

## Graphical abstract

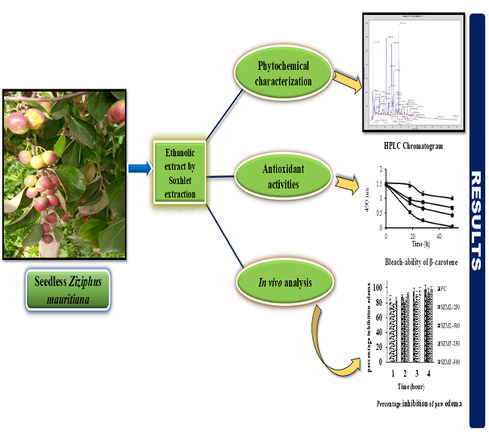

## Introduction

Inflammation is basically a biological response of the body that can be triggered by harmful stimuli to protect the body from infection and injury.^[1]^ It can be characterized by both acute and chronic forms. Acute inflammation is an immediate response after specific injury, results in localized responses such as oedema and leukocyte infiltration. However, if uncontrolled acute response persists for longer duration referred as chronic inflammation that triggers the production of cytokines.^[2]^ Chronic inflammation which is closely associated with the oxidative stress, leads to more severe conditions like diabetes, arthritis, cardiovascular and autoimmune diseases.^[3]^ Non‐steroidal anti‐inflammatory drugs (NSAIDs) are commonly used for their anti‐inflammatory properties.^[4]^ However, they are associated with adverse effects, various approaches have been explored to minimize these adverse effects underscoring the need for safer alternatives.^[5]^

Phytochemicals have attained great interest due to their great antioxidant and biological properties. Polyphenols which are secondary metabolites, abundant in fruits and vegetables having important health‐promoting effects including antioxidant, antimicrobial, anti‐inflammatory, anticancer and antihypertensive properties.[^6^, ^7^] Among these, phenolic acids and flavonoids like chlorogenic acid, gallic acid and coumarin have the promising reactive oxygen scavenging effects and inhibiting the inflammatory cytokines.^[8]^ Given their generally recognized as safe (GRAS) status, these natural products are increasingly preferred by consumers. Thus, there is need to explore natural products from overlooked and underutilized plant species, that can be utilized for potential pharmaceutical and nutraceutical application.^[9]^

Moreover, abrupt changes in climate during the last two decades in parallel with the rapid expansion in world's population (expected to be 9.6 billion in 2050) caused significant increases in food prices and boosted the land degradation.^[10]^ These factors are considered responsible for present food insecurity conditions especially in the underdeveloped countries. It has been estimated that in the next 35 years, the food demand will be 56 % more than today.^[11]^ So, in view of the present estimates the use of conventional food crops will not be enough to fulfill the world food requirements and there is an intense need to explore other non‐conventional food sources to fulfil the world food demand and to reduce pressure on conventional food crops.^[12]^

*Ziziphus mauritiana* Lam. (*Z. mauritiana*) belongs to the Rhamnaceae family, comprises approximately 100 species, and is suited to arid and semi‐arid climates.^[13]^ The fruit of this plant is highly valued in rural areas for its high nutritional content and is known as the “apple for the poor”. Traditionally, it has been used in treating various diseases in Pakistan and other countries including Afghanistan, Australia, India, Iran, and African countries.[^14^, ^15^] Furthermore, due to advancements in grafting techniques, researchers have developed seedless *Z. mauritiana* (SZM), commonly known as Sundari Apple Ber, using *Z. mauritiana* rootstocks with apple bud. The SZM fruit is sweet in taste with rudimentary seeds and have more flesh contents than conventional *Z. mauritiana* varieties, which are easier to eat and process as compared to the seeded varieties, having small size fruits with large seed. This variety offers enhanced nutritional quality and economic benefits.^[12]^ Its cultivation can be promoted by plantation on marginal farmland due to low maintenance and drought resistance. So, it could be a good candidate by its plantation for utilization of arid and semi‐arid regions in economic well beings. Sherani et al.^[12]^ grafted the local root stock of *Ziziphus mauritiana* with advanced scion cultivars to improve the quality and yield of fruits. However, the newly developed SZM species remains largely underutilized and neglected from a medicinal perspective.

To our knowledge, no sufficient data are available regarding the polyphenol contents, antioxidant and anti‐inflammatory potential of SZM leaf and fruit extracts. Therefore, this study was designed to evaluate the antioxidant and anti‐inflammatory properties of polyphenol‐rich extracts of seedless *Z. mauritiana*, focusing on the suppression of inflammatory biomarkers in the carrageenan‐induced rat paw edema model. The quantification of the polyphenols was performed using RP‐HPLC technique to identify the bioactive compounds from the polyphenol‐rich extracts responsible for these activities.

## Results and Discussion

### Yield of Extracts

The yield (g/100 g) of SZM leaf and fruit extracts is presented in Table 1. The ethanol leaf extract demonstrated a higher yield (19.2 g/100 g) than the fruit extract (17.2 g/100 g). There is non‐significant difference (*p*≥0.05) in the yield of both extracts. There is no report available in the literature that specifically reports the extract yield of SZM extracts. However, the yield of *Z. mauritiana* fruit extract in ethanol was reported by Delfanian et al.^[16]^ and that was 17.56–45.71 %. Dahiru et al.^[17]^ reported that leaf extract in ethanol gave 19.35 % yield.

**Table 1:** Yield, TPC, TFC and DPPH radical scavenging capacity of leaves and fruit extracts of seedless *Ziziphus mauritiana*.

Assays	Seedless *Ziziphus mauritiana* extracts		BHA
	Leaves	Fruits	
Yield (g/100 g) of dry plant material)	19.2±0.77^a^	17.2±0.86^a^	–
TPC (mg/100 g) of dry plant material, measured as gallic acid equivalent)	177.6±5.33^b^	137.8±5.51^a^	–
TFC (mg/100 g) of dry plant material, measured as catechin equivalent)	46.2±1.85^b^	14.1±0.71^a^	–
DPPH scavenging (%) by 10 μg/mL extract solution	63.5±3.81^b^	58.2±4.07^a^	68.2±2.73^c^

The given values are reported as mean±standard deviation of three independent experiments (n=3). Different alphabets in the same row represent significant differences (P≤0.05) among samples. Abbreviations: TPC, total phenolic content; TFC, total flavonoid content; DPPH, 2,2‐diphenyl‐1‐ picrylhydrazyl, butylated hydroxy anisole (BHA).

### Separation and Identification of Polyphenols by RP‐HPLC

**Figure 1:**
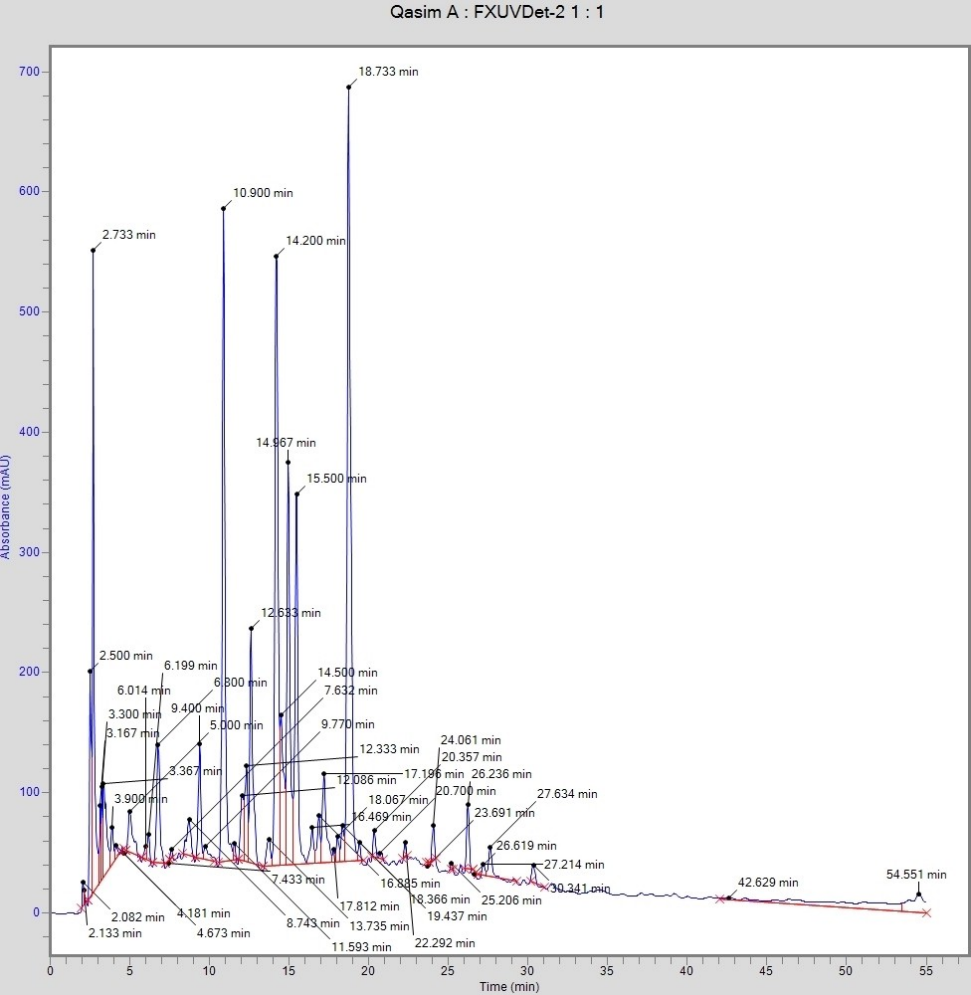
Typical chromatogram showing the separation of phenolic acids and flavonoids from seedless *Ziziphus mauritiana* leaves extract.

The composition of polyphenols of SZM leaf and fruit extracts was analyzed by RP‐HPLC (Table 2). A typical chromatogram demonstrating the separation of phenolic acid and flavonoid is presented in Figure 1. Twelve phenolic acids (chlorogenic acid, gallic acid, *p*‐coumeric, hydroxybenzoic acid, vanillic acid, caffeic acid, ferulic acid, salicylic acid, ellagic acid, sinapic acid, coumarin and benzoic acid) and four flavonoids (kaempferol, quercetin, rutin and myricetin) were identified in both leaf and fruit extracts of SZM. Chlorogenic acid (566.88 mg/kg) was abundantly found in leaf extract, followed by coumarin (517.77 mg/kg), quercetin (356.64 mg/kg) and ferulic acid (214.59 mg/kg) of dry plant material. The Leaf extract was rich in flavonoid contents, with myricetin, rutin, quercetin and kaempferol with concentrations 1.33, 50.54, 356.64 and 4.15 mg/kg, respectively. In fruit extract, again the major phenolic acid chlorogenic acid (1509.48 mg/kg), followed by salicylic acid (308.93 mg/kg) and gallic acid (210.24 mg/kg). The major flavonoids kaempferol and rutin were detected in the fruit extract, with concentrations of 10.02 and 21.52 mg/kg, respectively. A significant difference (*p*≤0.05) was noted in the concentration of most compounds between the leaf and fruit extracts.

**Table 2:** HPLC analysis for phenolic acids and flavonoids composition from seedless *Ziziphus mauritiana* leaves and fruit extracts.

Compounds	Retention time (min)	Concentration (mg/kg)	
		Leaf extract	Fruit extract
Chlorogenic acid	2.733	566.88±17.01^a^	1509.48±45.28^b^
*p*‐Coumeric acid	3.167	6.03±0.30^a^	11.82±0.47^b^
Gallic acid	3.300	198.35±7.93^a^	210.24±6.31^a^
Hydroxybenzoic acid	3.167	95.85±3.83^b^	26.98±1.08^a^
Caffeic acid	5.000	110.30±3.31^b^	10.61±0.53^a^
Vanillic acid	6.199	29.29±1.17^a^	46.36±1.85^b^
Kaempferol	6.800	4.15±0.17^a^	10.02±0.50^b^
Sinapic acid	8.743	56.69±2.27	—
Ferulic acid	10.900	214.59±6.44^b^	4.81±0.24^a^
Ellagic acid	13.735	69.01±2.76	—
Salicylic acid	14.200	1495.97±44.88	308.93±9.27^a^
Coumarin	14.967	517.77±20.71^b^	113.94±4.55^a^
Quercetin	15.500	356.64±10.60	—
Benzoic acid	18.733	14.42±0.58^a^	57.83±2.31^b^
Myricetin	20.357	1.33±0.07	—
Rutin	26.236	50.54±2.02^b^	21.52±0.86^a^

The given values are reported as mean±standard deviation of three independent experiments (n=3). Different alphabets in the same row represent significant differences (P≤0.05) among samples.

The analysis of complex plant extracts for phenolic compounds is typically performed on high‐performance liquid chromatography.^[18]^ Phenolic compounds are typically higher in leaves, owing to their abundance in colored tissues, while flavonoid compounds are prevalent in the epidermis and cuticle.^[18]^ Chlorogenic acid concentration found maximum in the fruit extract was comparable with the findings reported by Izu et al.,^[19]^ who reported that fruits are rich in chlorogenic acid and have significant antioxidant activity. No data in the literature available on the phenolic profiling of hybrid SZM leaf and fruit extracts. However, some studies reported in literature on the apple and *Z. mauritiana* fruit extracts. Kumari et al.^[20]^ reported that quercetin, chlorogenic acid, caffeic acid and hydroxy benzoic acid were the major phenolics in the apple extract. Yahia et al.^[21]^ mentioned rutin, quercetin, *trans* ferulic acid, chlorogenic acid, *p*‐coumaric in the fruit extract of *Z. mauritiana*. Ramar et al.^[22]^ reported quercetin, kaempferol, caffeic acid in the leaves extract of *Ziziphus mauritiana*. As SZM is grafted from red apple and *Z. mauritiana*, therefore the high concentration of major phenolics and flavonoids in SZM extracts might be attributed to its hybrid nature.

### Determination of TPC and TFC

In the present study total phenolic contents (TPC) and total flavonoid contents (TFC) of SZM leaf and fruit extracts were measured as shown in Table 1. The better results of TPC (177.6 mg/100 g) showed by the leaves extract than the fruit extract (137.8 mg/100 g) of dry plant material. The leaves extract also showed the better TFC (46.2 mg/100 g) than fruit extract (14.1 mg/100 g) of dry plant material. The results showed the significant difference (p≤0.05) among both the extracts.

Phenolic compounds are well known for the excellent antioxidant activity.^[23]^ There is a wide range of pharmacological and biochemical properties associated with phenolic and flavonoid compounds.^[24]^ Our results are consistent with the literature that *Ziziphus* leaves have high TPC as compared to other parts of the plant.[^25^, ^26^] No report is available in literature on TPC and TFC of SZM extracts. However, Yahia et al.,^[21]^ reported the higher TPC in leaf and fruit extracts of *Ziziphus mauritiana* with values of 532.95 mg GAE/100 g DW and 148.75 mg GAE/100 g DW and TFC 90.26 mg QE/100 g DW and 39.33 QE/100 g DW, respectively than reported in the present study. Parveen et al.^[27]^ also reported higher TPC (3.61 mg GAE/g) in the fruit extract of *Ziziphus mauritiana* than the SZM fruit extract of the present study. Javed et al.,^[28]^ reported the presence of 29.8 and 25.8 mg total phenolic/g of extract, measure as gallic acid equivalent in the pulp and leaves of *Z. mauritiana*. Kaur et al.,^[29]^ reported the flavonoid content in *Z. mauritiana* to be 119.31 mg/100 g. The variation in the TPC and TFC with the literature is due to the variation in the *Ziziphus* variety.

### DPPH Radical‐Scavenging Activity

The DPPH activity was performed to estimate the free radical scavenging of SZM leaf and fruit extracts as presented in Table 1. The highest radical scavenging activity (68.2 %) showed by the standard BHA. Among both extracts the leaves showed better radical scavenging activity (63.5 %) than was observed in the fruit extract (58.2 %). The results showed the significant difference (p≤0.05) in terms of the radical‐ scavenging potential.

DPPH radical scavenging activity method is concentration dependent, rapid and simple method, widely used for the evaluation of antioxidant activity.^[30]^ Polyphenols which are abundantly present as the secondary metabolites in the plant extracts protect the body against free‐radical damage.^[31]^ The leaf extract of SZM showed better radical‐scavenging activity in the present study due to its higher TPC than the fruit extract. There is not any specific report available in literature on DPPH radical‐scavenging activity of SZM leaf and fruit extracts. However, our results are comparable with the findings of Riaz et al.^[18]^ who observed 62.5 % and 57.7 % inhibition of DPPH with leaf and fruit extracts of *Z. mauritiana*, respectively. Kaur et al.,^[29]^ reported 74.84 % radical scavenging activity of *Ziziphus mauritiana* extract that is higher than reported in the present study.

### Bleachability of β‐carotene Assay

**Figure 2:**
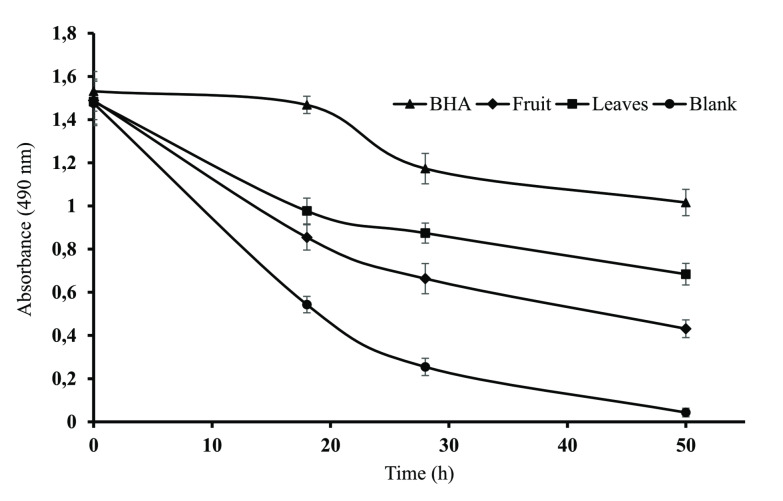
Antioxidant activity of Seedless *Ziziphus mauritiana* leaves and fruits extracts in term of bleach‐ability of β‐carotene‐linoleic acid emulsion. Values are reported as mean±standard deviation of triplicate determinations (n=3).

The bleachability of β‐carotene serves as an indicator of the antioxidant activity of SZM as depicted in Figure 2. The leaf extract exhibited a smaller decrease in absorbance, suggesting better antioxidant activity than the fruit extract. When the results were compared with standard BHA, the order of activity observed was as follows; BHA>SZM leaves extract>SZM fruits extract>blank.

For evaluating the antioxidant potential of different extracts utilizing multiple assays provides comprehensive understanding, as each assay targets the specific aspect of the antioxidant activity. Several studies have reported the role of polyphenol extracts in enhancing antioxidant capacity.[^32^, ^33^] The SZM extracts showed better antioxidant activity that might be attributed to the phenolic acids and flavonoids within these extracts.^[34]^ However, there are no existing results of bleachability of β‐carotene in the linoleic acid system available in literature for SZM leaf and fruit extract against which we can directly compare our findings.

### *In Vivo* Acute Inflammatory Model

**Figure 3:**
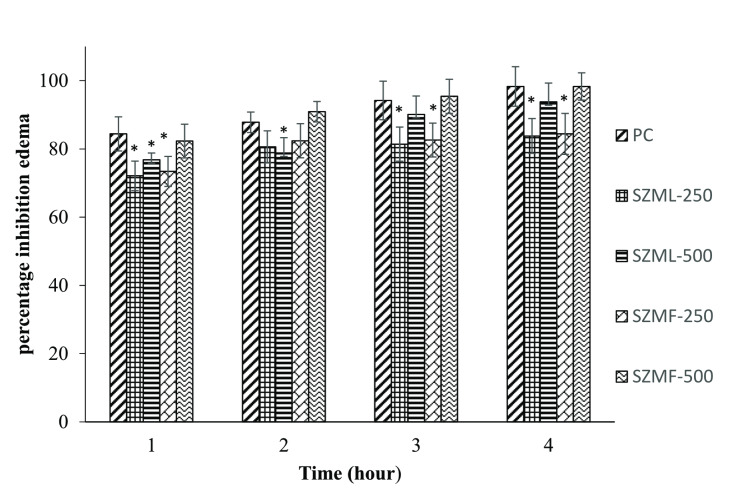
Percentage inhibition of paw edema in carrageenan induced rat model by different treatment groups over 1 to 4 hours. The given values are reported as mean±standard deviation of 18 observations (n=3×6=18). * Showed significant (p0.05) difference from PC group. SZML‐250 (Seedless *Z. mauritiana* leaves extract 250 mg/kg body weight); SZML‐500 (Seedless *Z. mauritiana* leaves extract 500 mg/kg body weight); SZMF‐250 (Seedless *Z. mauritiana* fruit extract 250 mg/kg body weight); SZMF‐500 (Seedless *Z. mauritiana* fruit extract 500 mg/kg body weight).

The paw diameter (mm) data for assessing the anti‐inflammatory activity of SZM leaves and fruit are shown in Table 3. The mean paw diameter (mm) was found to be non‐significant across all groups at zero hour. However, after one hour the maximum inflammation was observed in all groups post oral administration of ethanolic extract. Two hours, post‐administration, the inflammation was decreased in all treatment groups as compared to the normal control (NC) group. The most significant reduction was noted in SZMF‐500 group, which was comparable to the PC group. After three‐ and four‐hours post‐administration, inflammation continued to decrease significantly in SZMF‐500 group compared to all other treatment groups, while remaining comparable to the PC group. The percentage inhibition over a 1 to 4 h period is illustrated in Figure 3. The SZMF‐500 group showed inhibition rate (98.1 %), which was comparable to the PC group (98.4 %). This was followed by SZML‐500 group (93.8 %), the SZMF‐250 group (84.4 %) and SZML‐250 group (83.8 %).

**Table 3:** Rats paw diameter of carrageenan‐induced rat model of all treatment groups.

Groups	Paw Diameter (mm)				
	0 hour	1 hour	2 hours	3 hours	4 hours
DC	5.15±0.36^a^	6.62±0.39^b^	6.80±0.41^b^	6.87±0.41^b^	6.94±0.42^b^
PC	4.98±0.35^a^	5.21±0.31^a^	5.18±0.36^a^	5.08±0.36^a^	5.01±0.35^a^
SZML‐250	5.06±0.30^a^	5.47±0.38^a^	5.41±0.32^a^	5.38±0.38^a^	5.35±0.32^a^
SZML‐500	4.98±0.34^a^	5.32±0.32^a^	5.27±0.37^a^	5.15±0.31^a^	5.09±0.31^a^
SZMF‐250	5.02±0.30^a^	5.41±0.33^a^	5.35±0.37^a^	5.32±0.37^a^	5.30±0.32^a^
SZMF‐500	5.01±0.35^a^	5.27±0.32^a^	5.16±0.36^a^	5.09±0.31^a^	5.04±0.35^a^

The given values are reported as mean±standard deviation of 18 observations (n=3×6=18). Different alphabets in the same column represent significant differences (P≤0.05) among different groups. Abbreviations: DC (Disease control carrageenan 0.1 ml of 1 % w/v carrageenan solution); PC (Positive control indomethacin 10 mg/kg body weight); SZML‐250 (Seedless *Z. mauritiana* leaves extract 250 mg/kg body weight); SZML‐500 (Seedless *Z. mauritiana* leaves extract 500 mg/kg body weight); SZMF‐250 (Seedless *Z. mauritiana* fruit extract 250 mg/kg body weight); SZMF‐500 (Seedless *Z. mauritiana* fruit extract 500 mg/kg body weight).

The carrageenan‐induced model serves as a well‐established method for studying acute inflammation. Polyphenols are known for their potential antioxidant and anti‐inflammatory properties.^[35]^ Xu et al.^[36]^ reported that polyphenols, especially chlorogenic acid could show anti‐inflammatory activity. In the present investigation, chlorogenic acid potentially responsible for the remarkable anti‐inflammatory activity of SZM fruit extract at a dose level of 500 mg/kg BW, as it is identified as a major compound. Similarly, rutin and quercetin, which are known to have anti‐inflammatory activity, were detected in SZM extracts, suggesting their contribution to the potent anti‐inflammatory effect.^[37]^ While no reports specifically addressing the anti‐inflammatory activity of SZM extracts are available, Alsyari and Wahab^[38]^ reported the anti‐inflammatory effect of other *Ziziphus* species. Additionally, Mesaik et al.^[39]^ demonstrated the anti‐inflammatory effect of ethanolic fruit extract of *Z. mauritiana*. The results indicate that SZM fruit extract could be a promising candidate for developing new anti‐inflammatory therapies.

### Determination of Inflammatory Cytokines

The results of proinflammatory cytokines i. e. Interleukin‐6 (IL‐6), Tumor necrosis factor‐α (TNF‐α), and C‐reactive protein (CRP) levels are presented in Table 4. Both the leaf and fruit extracts significantly reduced the secretions of inflammatory mediators in serum as compared to the DC group. IL‐6 and TNF‐α levels were 869.45 pg/mL and 644.44 pg/mL, respectively in DC group that were significantly higher (p≤0.05) then NC and treatment groups. Administration of SZM fruit extract at 500 mg/kg BW (SZMF‐500 group) showed significantly (p≤0.05) reduced IL‐6 secretion to 280.56 pg/mL comparable to the standard drug indomethacin (277.78 pg/mL), and TNF‐ α level to186.11 pg/mL, close to indomethacin (188.89 pg/mL). Carrageenan injection elevated the CRP level in the DC group (1.24 mg/L) compared to NC group (0.80 mg/L). The treated groups prevented the elevation of CRP level, and the best effect was showed by SZMF‐500 (0.84 mg/L) comparable to the PC group (0.83 mg/L).

**Table 4:** Effect of SZM extract on inflammatory cytokines secretions in serum of carrageenan induced rat model.

Groups	IL‐6 (pg/mL)	TNF‐α (pg/mL)	CRP (mg/L)
NC	194.44±11.67^[a]^	166.67±10.02^[a]^	0.80±0.05^[a]^
DC	869.45±52.17^[b]^	644.44±38.67^[b]^	1.24±0.07^[b]^
PC	277.78±16.67^[a,b]^	188.89±11.33^[a]^	0.83±0.06^[a]^
SZML‐250	372.15±22.34^[a,b]^	275.29±16.52^[a,b]^	0.92±0.05^[a,b]^
SZML‐500	298.13±17.89^[a,b]^	192.31±11.54^[a,b]^	0.86±0.04^[a]^
SZMF‐250	342.16±20.53^[a,b]^	265.12±15.91^[a,b]^	0.88±0.05^[a]^
SZMF‐500	280.56±16.83^[a,b]^	186.11±11.17^[a]^	0.84±0.07^[a]^

The given values are reported as mean±standard deviation of 18 observations (n=3×6=18). ^[a]^ Showed significant difference (P≤0.05) with DC group and ^[b]^ represented significant difference (P≤0.05) with NC group. Abbreviations: IL‐6 (Interleukin‐6), TNF‐α (Tumor necrosis factor‐α), CRP (C‐reactive protein); DC (Disease control carrageenan 0.1 mL of 1 % w/v carrageenan solution); PC (Positive control indomethacin 10 mg/kg body weight); SZML‐250 (Seedless *Z. mauritiana* leaves extract 250 mg/kg body weight); SZML‐500 (Seedless *Z. mauritiana* leaves extract 500 mg/kg body weight); SZMF‐250 (Seedless *Z. mauritiana* fruit extract 250 mg/kg body weight); SZMF‐500 (Seedless *Z. mauritiana* fruit extract 500 mg/kg body weight).

The inflammatory markers are indicative of inflammatory processes in the body because they are primary indicator of inflammation in the body and failure to eliminate acute responses can lead to chronic conditions. The level of these biomarkers rises significantly in response to carrageenan induction. Abnormal levels of these inflammatory markers are associated with various diseases.^[40]^ C‐reactive protein (CRP) is an acute‐phase protein as its level increases rapidly in both acute and chronic inflammation and also a key factor in monitoring inflammation.^[41]^ Interleukin (IL‐6) play a crucial role in coordinating with different immune cells and play significant role in acute phase response.^[42]^ The reduction in the level of anti0inflammatory markers indicates a positive response to treatment. Chlorogenic acid in plant extracts is reported as an anti‐inflammatory agent.^[43]^ SZMF‐500 group showed the maximum reduction of CRP, IL‐6 and TNF‐α levels that may be due to its high concentration of chlorogenic acid and its synergistic effects with other polyphenols. No data is available in the literature regarding to the suppression of proinflammatory cytokines by SZM extracts, although Mohankumar et al.^[44]^ reported that *Z. mauritiana* lowered the elevated IL‐6 and TNF‐α levels.

### Oxidative Stress Parameters

The effects of SZM extracts on endogenous oxidative enzymes are presented in Table 5. Levels of SOD (4.34 μg/mg), CAT (6.06 mmol/mg), GPx (5.12 μg/mg) and GSH (2.93 μg/mg) were significantly (*p*≤0.05) decreased in the DC group compared to the NC group, indicating the development of oxidative stress in the animals. However, the level of MDA (0.80 μmol/mg) in the liver homogenate was found to be higher than in the NC group (0.24 μmol/mg) and PC group (0.46 μmol/mg), thus indicating oxidative stress. Treatment with SZM extracts and standard indomethacin increased the level of all oxidative enzymes and showed a better protective effect in response to oxidative stress. Similarly, the MDA level is reduced in all the treatment groups except in the DC group due to oxidative stress. The leaf extract at a dose level of 500 mg/kg caused a significant change in antioxidant enzyme activity: SOD (6.98 μg/mg), CAT (8.72 mmol/mg), MDA (0.42 μmol/mg), GPx (6.88 μg/mg) and GSH (4.35 μg/mg), showing better protective effect than all other treated groups and positive control indomethacin: SOD (5.97 μg/mg), CAT (8.37 mmol/mg), MDA (0.46 μmol/mg), GPx (6.95 μg/mg) and GSH (4.37 μg/mg).

**Table 5:** Effect of SZM on oxidative stress parameters of different treatment groups of carrageenan induced rat model.

Groups	SOD (μg/mg)	CAT (mmol/mg)	MDA (μmol/mg)	GPx (μg/mg)	GSH (μg/mg)
NC	6.92±0.42^[a]^	9.64±0.58^[a]^	0.24±0.01^[a]^	7.21±0.43^[a]^	4.48±0.27^[a]^
DC	4.34±0.26^[b]^	6.06±0.36^[b]^	0.80±0.05^[b]^	5.12±0.31^[b]^	2.93±0.18^[b]^
PC	5.97±0.36^[a,b]^	8.37±0.50^[a,b]^	0.46±0.03^[a,b]^	6.95±0.42^[a]^	4.37±0.26^[a]^
SZML‐250	5.15±0.31^[a,b]^	8.08±0.48^[a,b]^	0.52±0.04^[a,b]^	6.23±0.37^[a,b]^	3.39±0.20^[a,b]^
SZML‐500	6.98±0.42^[a]^	8.72±0.52^[a]^	0.42±0.03^[a,b]^	6.88±0.41^[a]^	4.35±0.26^[a]^
SZMF‐250	5.81±0.38^[a,b]^	8.34±0.54^[a,b]^	0.50±0.02^[a,b]^	5.95±0.36^[a,b]^	3.18±0.19^[a,b]^
SZMF‐500	5.88±0.35^[a,b]^	8.65±0.56^[a]^	0.48±0.06^[a,b]^	6.35±0.38^[a,b]^	4.32±0.26^[a]^

The given values are reported as mean±standard deviation of 18 observations (n=3×6=18). ^[a]^ Showed significant difference (P≤0.05) with DC group and ^[b]^ represented significant difference (P≤0.05) with NC group. Abbreviations: DC (Disease control 0.1 ml of 1 % w/v carrageenan solution); PC (Positive control indomethacin 10 mg/kg body weight); SZML‐250 (Seedless *Z. mauritiana* leaves extract 250 mg/kg body weight); SZML‐500 (Seedless *Z. mauritiana* leaves extract 500 mg/kg body weight); SZMF‐250 (Seedless *Z. mauritiana* fruit extract 250 mg/kg body weight); SZMF‐500 (Seedless *Z. mauritiana* fruit extract 500 mg/kg body weight); SOD (superoxide dismutase); CAT (catalase); MDA (malondialdehyde); GPx (glutathione peroxidase); GSH (reduced glutathione).

Inflammation causes the overproduction of reactive oxygen species (ROS) which are responsible for oxidative stress and harmful to living organisms.^[45]^ Oxidative stress is frequently measured by assessing oxidative stress markers, such as Superoxide Dismutase (SOD), Catalase (CAT), Malondialdehyde (MDA), Glutathione Peroxidase (GPx) and Glutathione (GSH) against the production of free radicals.^[46]^ By monitoring these parameters, the extent of oxidative stress in the body can be estimated. The oxidative stress markers decreased significantly in the disease control group except MDA, showing oxidative stress in the treated rats. SOD plays a main role in neutralizing a superoxide radical and a quick responder against the oxidative stress.^[47]^ CAT enzymes work rapidly in converting hydrogen peroxide into water and oxygen, which are produced as byproducts when cells produce energy.^[48]^ GPx is an antioxidant enzyme that plays a role in neutralizing hydrogen peroxidase in water and lipid peroxidase.^[48]^ GSH is known to be a master antioxidant; it can directly neutralize free‐radicals.^[49]^ MDA is produced as a byproduct of lipid peroxidation and its level rises in response to oxidative stress, leading to various health issues.^[50]^ Polyphenolic compounds, abundant in plant extracts play a crucial role in cellular protection against oxidative cell‐induced injury.^[51]^ Flavonoids effectively counteract oxidative stress by inhibiting the peroxidation of fatty acids in cell membranes.^[52]^ Dahiru et al.^[53]^ reported the effective role of *Ziziphus mauritiana* extract in normalizing the level of antioxidant enzymes in a rat model. In our study, the leaf extract of SZM exhibited a remarkable antioxidant response, especially at a high dose level of 500 mg/kg BW followed by the fruit extract of SZM at 500 mg/kg BW that might be attributed to its polyphenolic compounds. These compounds act synergistically to protect against oxidative damage, such as direct scavenging of ROS which is highly responsible for oxidative stress. Therefore, SZM extracts hold promising therapeutic potential for combating oxidative stress‐related disorders.

## Conclusions

The study results indicate that both the leaf and fruit extracts of seedless *Ziziphus mauritiana* (SZM) exhibit potential antioxidant and remarkable anti‐inflammatory activities. These benefits are demonstrated by the reduction of proinflammatory cytokines and oxidative stress. Notably, the high concentration of chlorogenic acid, gallic acid, kaempferol, quercetin, rutin and myricetin detected by Rp‐HPLC proved the potential of the SZM as a natural antioxidant and anti‐inflammatory agent. *In vitro* assays revealed that SZM leaf and fruit extracts possess high antioxidant activities, as measured by TPC, TFC, DPPH radical scavenging and bleachability of β‐carotene bleaching assays. Given these properties, SZM shows promise as a non‐conventional food source and natural medicine for anti‐inflammatory purposes. The large‐scale cultivation of this seedless *Z. mauritiana* based on its adaptability and high nutritional values is highly recommended in salt effected coastal areas. Further research should be carried out for isolation of phenolic compounds for potential medicinal uses. The investigation at cellular and molecular levels and advances in gene editing hold a potential to revolutionize the medicine. It will also provide additional scientific evidence for the therapeutic use of SZM leaves and fruit in treating inflammation after further clinical trials.

## Experimental Section

### SZM Grafting, Sample Collection and Authentication

Leaves and fruits of grafted SZM (Sundari apple ber) plants were collected during the month of February‐March 2022 from the plants cultivated in a research fields of Botanical Garden (latitude 30° 30′ N, longitude 73° 10′ E and altitude 213 m), Government College University Faisalabad, Pakistan. This grafted species was developed through a two‐step grafting process: red apple stems were initially grafted on root stock of *Z. mauritiana* Lam (Chinese jujube) followed by grafting the resulting rootstock with the stem of the red apple. The specimens were further authenticated by Dr. Qasim Ali, Associate Professor and Convener, Botanical Garden, Department of Botany with the voucher number (380‐Bot‐23), Herbarium, Government College University Faisalabad, Pakistan.

### Reagents, Standards and Chemicals

All the reagents and standards, including linoleic acid, gallic acid, catechin, Folin‐Ciocalteu, carrageenan, butylated hydroxy anisole (BHA), sodium azide, trichloroacetic acid, ethylenediamine acetic acid, thio‐barbituric acid and all other chemicals, reagents used in studies were collected form Sigma‐Aldrich Co (St Louis, USA, MO).

### Pretreatment and Extract Preparation

Leaves and fruits of SZM were washed, dried, and powdered to 80 mesh size using an electric grinder. The extraction was performed using a Soxhlet apparatus (500 mL capacity) with absolute ethanol, following the method reported by Jha and Sit.^[54]^ The extracted solution was evaporated using a rotary evaporator. The yield was calculated by given formula in equation 1.

(1) $$Yield\;\left(\frac{g}{100g}\right)=\frac{weight\;of\;dry\;extract}{weight\;of\;dry\;plant\;material\;}\times 100$$

### Separation and Identification of Polyphenols by RP‐HPLC

The separation and identification of polyphenols in SZM leaf and fruit extracts (10 mg/mL) were conducted by RP‐HPLC, following the method previously reported by Khanam et al..^[23]^ The HPLC system (Perkin Elmer, Shelton, CT, USA) connected with UV/Visible detector and Hypersil GOLD C_18_ column (internal diameter measured 250×4.6 mm, particle size of 5 μm) along with a guard column. Concentration versus peak area plots were generated to obtain a standard curve. The quantification of the sample was done following the external standard method. For identification of analytes, the samples were spiked, and their retention times were compared with the standards.

### Antioxidant Assays

#### Determination of Total Phenolics and Flavonoid Contents

Folin–Ciocalteu method was assessed to measure the total phenolic contents (TPCs), while the total flavonoid contents (TFCs) were assessed by using aluminium chloride method, following the procedure described by Hussain et al..^[34]^ Calibration curves with different concentrations of gallic acid (10–320 ppm) and catechin (10–160 ppm), resulting in equations Y=9.1756x+0.001 and Y=1.1923x+0.0035, respectively. TPCs (mg/100 g) expressed as gallic acid equivalent, and TFCs (mg/100 g) expressed as catechin equivalent of dry plant material.

#### Determination of DPPH Radical Scavenging Activity

The radical scavenging activities of SZM leaf and fruit extracts were assessed using the stable free‐radical DPPH, as provided method Hussain et al..^[34]^ Briefly, extract concentrations of 10 μg/mL and BHA prepared in analytical grade methanol and then mixed with 2 mL of DPPH solution (90 μmol). Finally, the percentage scavenging of DPPH was estimated at 517 nm using a spectrophotometer.

#### Bleachability of β‐Carotene Assay

The β‐carotene bleaching assay was conducted following the method.^[55]^ Briefly, the β‐carotene (0.5 mg) was mixed with linoleic acid (1000 mg), tween 40 (100 mg) and chloroform (1 mL) for preparing the stock solution. The chloroform was evaporated from the mixture using rotary evaporator. After incubating the mixture, the absorbance was recorded at 490 nm for up to 50 hours. BHA was run as a positive control, and an emulsion without any antioxidant served as the oxidation control.

### Acute Anti‐Inflammatory Model

#### Experimental Animals

The male Wistar rats (weighing 140–170 g) collected from the animal house of GC University, Faisalabad. *In vivo* experiments were initiated after acclimatizing the animals for one week under standard conditions (ambient humidity 40–60 %, 27 °C±2 °C temperature) with free availability of water and food.^[23]^

#### Ethics Approval

The experiments were conducted after obtaining approval from the Institutional Review Board (Reference. No. GCUF/ERC/238).

#### Study Design

Male Wistar rats were divided into five groups, each group having six rats (n=6). The normal control (NC) group (received no specific treatment). The disease control (DC) group (received an injection 0.1 mL (1 %) of freshly prepared carrageenan). The positive control (PC) group (received a standard dose of indomethacin 10 mg/kg body weight). The treatment groups included SZML‐250 (received Seedless *Ziziphus mauritiana* leaves extract 250 mg/kg body weight) SZML‐500 (received Seedless *Z. mauritiana* leaves extract 500 mg/kg body weight), SZMF‐250 (received Seedless *Z. mauritiana* fruit extract 250 mg/kg body weight), and SZMF‐500 (received Seedless *Z. mauritiana* fruit extract 500 mg/kg body weight).

After 14 days of the relevant treatments to all groups, the inflammation was induced by injecting carrageenan (0.1 mL) below the plantar fascia of the right hind paw of rats in all groups except NC group. Paw swelling was measured using a digital vernier caliper for 4 h, and then by using DC group as a standard the percentage inhibition was calculated.^[5]^

#### Collection of Blood and Liver

After completing the experiment, the animals were sacrificed by complete anesthesia using pentothal injection. Blood was collected in plain bottles, centrifuged at about 3000 rpm for 15 minutes to separate the serum. Livers were collected and placed in a 10 % formalin solution.

#### Inflammatory Cytokines

Inflammatory cytokines were assayed in serum including interleukin‐6 (IL‐6) using an ELISA kit (Cat#: Y‐84561‐48 T Wuhan, China) and tumor necrosis factor‐α (TNF**‐ *α*)** was assayed by using ELISA kit (Cat# Y‐83079‐48 T Wuhan, China) with the manufacturer's recommendations.^[56]^ The standard curves were plotted by using standard cytokines for calculating the concentrations of unknown samples. C‐reactive protein (CRP) level was estimated in serum from commercially available ELISA kit.^[57]^

#### Oxidative Stress Parameters

The livers were excised and 1 g of each liver was homogenized by using an automated tissue homogenizer in phosphate buffer (pH 7.4) as reported by Saleem et al..^[56]^ For the estimation of oxidative stress, the homogenate after centrifugation was stored at – 20 °C. Further the protein contents were estimated by the method followed by Saleem et al..^[56]^

#### Estimation of Superoxide Dismutase (SOD) Activity

The SOD activity was performed according to the reported method Saleem et al..^[58]^ Briefly, the liver homogenate (0.1 mL) was mixed with potassium phosphate buffer (0.1 M) and then 0.1 mL of pyrogallol solution (1 M) was added in the mixture. Finally, the absorbance was taken at 325 nm by spectrophotometer and then correlated with the standard curve.

#### Estimation of Catalase (CAT) Activity

The CAT activity was determined by the reported method Saleem et al..^[58]^ For this study, the liver homogenate (0.05 mL) mixed with phosphate buffer (50 Mm, pH 7.4) and H_2_O_2_ (30 mM, 1 mL). The reaction mixture optical density was taken at 240 nm. The CAT activity was calculated by given formula given in equation 2.

(2) $$CAT\;activity=\;\frac{OD}{E}\;\times volume\;of\;sample\;\left(mL\right)\;\times mg\;of\;protein$$

Where OD is the optical density, E is the Extinction coefficient 1.56×10^5^

#### Estimation of Malondialdehyde (MDA) Activity

The MDA activity was calculated by the method described by Bhangale and Acharya.^[59]^ Briefly, for the assessment, liver homogenate (1 mL) was combined with trichloroacetic acid (TCA), 0.25 M HCl and thio‐barbituric acid (TBA). After centrifugation about 10 min at 4000 rpm the absorbance was taken as 532 nm. The concentration of the MDA level was computed by following the formula given below in equation 3.

(3) $$MDA\;concentration=\;\frac{Absorbance\;\times 100\;\times total\;voume}{E\;\times liver\;weight\;\times volume\;of\;aliquots}$$

Where, E= is the molar extinction co‐efficient 1.56×10^5^.

#### Estimation of Glutathione Peroxidase (GPx) Activity

For assessment of GPx activity, 0.1 mL of liver homogenate was mixed with ethylenediamine tetra acetic acid (EDTA) and sodium azide. The termination of reaction was done by adding trichloroacetic acid. The separated upper layer after centrifugation at 2000 rpm mixed with 4 mL of disodium hydrogen phosphate and 0.5 mL of 5,5‐dithiobis‐2‐nitrobenzoic acid (DTNB). Finally, the absorbance of reaction mixture was noted at 420 nm.^[59]^

#### Estimation of Glutathione (GSH) Activity

The activity of GSH was determined with the previously described method.^[59]^ Briefly, liver homogenate (1 mL) was mixed with 10 % trichloroacetic acid which was then centrifuged at 3000 rpm for 30 min. The separated supernatants were mixed with the buffer solution of sodium phosphate and DTNB. The absorbance of the reaction mixture was absorbed at 412 nm by spectrophotometer. GSH was determined by the following formula given in equation 4.

(4) $$GSH=Absorbance-\frac{0.00314}{0.034}\;\times \;\frac{Dilution\;factor}{Liver\;homogenate}\;\times Volume\;of\;aliquot\;$$

#### Statistical Analysis

The statistical analysis was used to evaluate the data in triplicates and reported as mean standard deviation (SD). STATISTICA 5.5 (Stat Sift Inc, Tulsa, USA) software was used to apply the one‐way analysis of variance (ANOVA) followed by hoc testing. Statistically significant differences (*p*≤0.05) between the means were observed.

## Author Contributions

Concept and research supervision: LN and AIH; Methodology: AIH, HAR; Data collection and preparation of first draft: AK; Resources: AIH, HAR; Revision and refining of the research article: EHM, HAR and LN.

## Conflict of Interests

The authors declare no conflict of interest.

1

## Data Availability

The data will be provided by the corresponding author when required.
